# In situ decorated pd NPs on Triazin-encapsulated Fe_3_O_4_/SiO_2_-NH_2_ as magnetic catalyst for the synthesis of diaryl ethers and oxidation of sulfides

**DOI:** 10.1038/s41598-024-75681-x

**Published:** 2024-10-25

**Authors:** Durgesh Singh, Kamini Singh, Pawan Sharma, Yashwantsinh Jadeja, Johar MGM, Priyanka Singh, Kiranjeet Kaur, M. Atif, Mohammed A. El-Meligy, Beneen Husseen

**Affiliations:** 1 Department of Chemistry, School of Chemical Sciences and Technology, Dr.Harisingh Gour Vishwavidyalaya (A Central University), Sagar, 470003 India; 2 Department of Chemistry, Deen Dayal Upadhyay Gorakhpur University, Gorakhpur, 273009 India; 3 Department of Chemistry, School of Sciences, Jain (Deemed-to-be) University, Bengaluru, 560069 Karnataka India; 4 Department of Sciences, Vivekananda Global University, Jaipur, Rajasthan, 303012 India; 5 Marwadi University Research Center, Department of Chemistry, Faculty of Science, Marwadi University, Rajkot, 360003 Gujarat India; 6 Management and Science University, Shah Alam, Selangor Malaysia; 7 NIMS School of Allied Sciences and Technology, NIMS University, Rajasthan, Jaipur, 303121 India; 8 Chandigarh Pharmacy College, Chandigarh Group of colleges-Jhanjeri, Mohali, 140307 Punjab India; 9 Department of Physics and Astronomy, College of Science, King Saud University, P O Box 2455, Riyadh, 11451 Saudi Arabia; 10 Jadara University Research Center, Jadara University, P O Box 733, Irbid, Jordan; 11 Applied Science Research Center, Applied Science Private University, Amman, Jordan; 12 Medical laboratory technique college, the Islamic University, Najaf, Iraq; 13 Medical laboratory technique college, the Islamic University of Al Diwaniyah, Al Diwaniyah, Iraq

**Keywords:** Complex, Sulfides, Triazin, Ether, Fe_3_O_4_, Chemistry, Catalysis, Green chemistry, Physical chemistry

## Abstract

This article is devoted to the synthesis of a new magnetic palladium catalyst that has been immobilized on A-TT-Pd coated-magnetic Fe_3_O_4_ nanoparticles. Such surface functionalization of magnetic particles is a promising method to bridge the gap between heterogeneous and homogeneous catalysis approaches. The structure, morphology, and physicochemical properties of the particles were characterized through different analytical techniques, including TEM, FT-IR, XRD, SEM, EDS, TGA-DTG, ICP, and VSM techniques. The obtained Fe_3_O_4_@SiO_2_@A-TT-Pd performance can show excellent catalytic activity for the synthesis of diaryl ethers and oxidation of sulfides, and the corresponding products were obtained with high yields. The advantages of this catalyst include a simple test method, green reaction conditions, no use of dangerous solvents, short reaction time, low catalyst loading, and reusability. Also, the nanocatalyst was easily separated from the reaction mixture with the help of a bar magnet and recovered and reused several times without loss of stability and activity.

**Supplementary Information:**

The online version contains supplementary material available at 10.1038/s41598-024-75681-x.

## Introduction

Nanomaterials that can be magnetically separated are considered one of the most significant material classes due to their distinct physicochemical properties. They have garnered interest from a diverse group of researchers^1,2^. With their potential as green heterogeneous catalysts in diverse organic functional group transformations and as catalytic supports, spinel ferrite compounds show great promise for use in industry and technology^3^. Over the last ten years, magnetic nanoparticles have been extensively used as a support in the production of magnetic nanocatalysts due to their simple preparation and easy retrieval using a magnetic field^4^. The easy separation of these nanoparticles from the reaction mixture using an external magnet is one of the benefits of magnetic nanoparticles^5^. The field of catalysis science plays a central role in numerous crucial organic reactions. Many essential organic functional group transformations necessitate the presence of a catalyst in the reaction environment to enable the selective conversion of reagents and synthons into the desired products with high efficiency^6^. The use of diverse nanomaterials as catalysts has globally captivated attention because of their distinctive ability to transform manufacturing processes into environmentally friendly, more sustainable, and cost-effective methods^7^. Among heterogeneous catalysts, Fe_3_O_4_ has been greatly favored since they are simply synthesized and surface-modified using a magnet^8,9^. Different catalysts can be supported on Fe_3_O_4_ nanoparticles due to easy separation after several reuses. Over the past decade, scientists have achieved a significant milestone with the discovery of the C-O coupling reaction facilitated by palladium-containing complexes^10,11^. Transition metal catalyst systems have greatly transformed the synthesis of organic structures through carbon-heteroatom coupling reactions^12–14^. These model reactions are extensively utilized in the synthesis of pharmaceutical, agricultural, and chemical compounds^15–17^. Diaryl ethers are ubiquitous in targeted synthetic and natural products, including agricultural and pharmaceutical chemicals, pharmaceuticals, fragrances, and flavorings^18,19^.

The targeted oxidation of organic sulfur compounds holds significant importance in biological, synthetic, and industrial contexts^20^. Sulfides play crucial roles in both biological and industrial processes^21^. They serve as significant reagents in organic synthesis, acting as protective agents for thiol, facilitating sulfonylation of enolates and other anions, and enabling the synthesis of organo-sulfur compounds through C–S bond formation^22^. Moreover, they are essential starting materials for the preparation of sulfenyl and sulfinyl reagents^23^. The selective oxidation of sulfides to sulfoxides holds great significance in organic chemistry^24^. Some biologically active sulfoxides are crucial as therapeutic agents, serving as antifungal, antibacterial, anti-atherosclerotic, anti-ulcer, antihypertensive, psychotropic, and vasodilator compounds^25^. A variety of methods, such as different catalysts and various oxidants, have been employed for the selective oxidation of organic sulfides and thiols. In this context, the use of H_2_O_2_ as a green oxidant has garnered significant interest due to its environmental implications, accessibility, high atom efficiency, and relatively lower costs compared to other oxidizing agents^26^.

Considering the interesting benefits of heterogeneous catalysts with the use of novel and green materials, herein, we reported the synthesis of an efficient and heterogeneous novel A-TT-Pd coated on Fe_3_O_4_ MNPs and its application in the synthesis of diaryl ethers and oxidation of sulfides in high yields under mild conditions.

## Experimental

### Preparation of Fe_3_O_4_@SiO_2_@A-TT-Pd

**Scheme 1:**
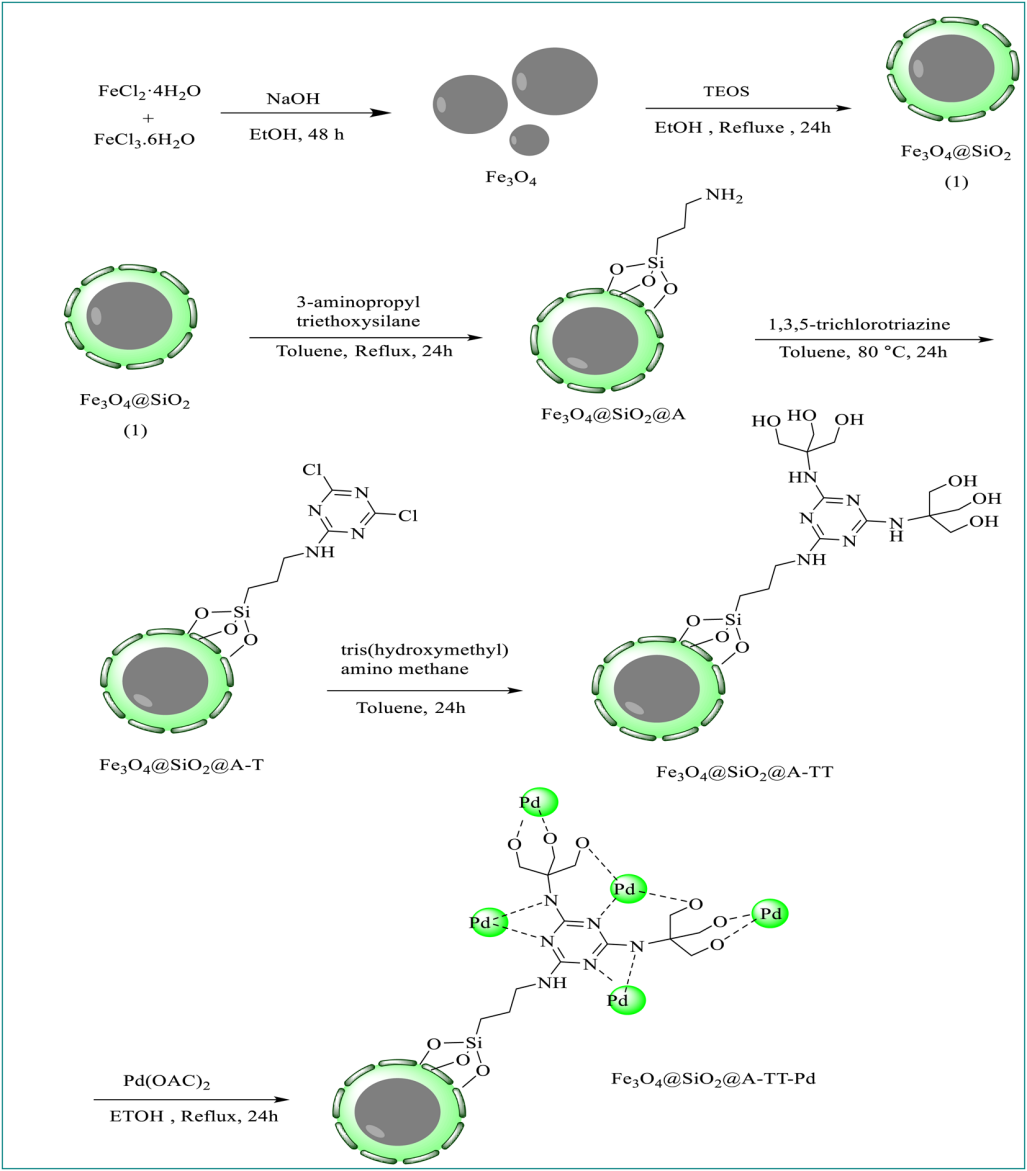
Schematic diagram of Fe_3_O_4_@SiO_2_@A-TT-Pd.

For the synthesis of Fe_3_O_4_ nanoparticles, a mixture of FeCl_2_.3H_2_O (2 g) and FeCl_3_.6H_2_O (4 g) in 25 mL ethanol at room temperature was added to a round bottom flask. After adding 2 g of NaOH to the reaction container, the solution was vigorously stirred at 80 °C for 48 h. Subsequently, the prepared magnetic nanoparticles were separated using a magnet, washed with deionized water, and dried at 60 °C for 10 h. Following this, 1 gram of the obtained Fe_3_O_4_ was dispersed in a mixture of 40 mL of ethanol, 5.0 mL of ammonia solution, and 20 mL of H_2_O. Then, 2 g of PEG-400 and 3 mL of tetraethyl orthosilicate (TEOS) were added to the mixture, which was stirred for 24 h at room temperature. The resulting product (Fe_3_O_4_@SiO_2_) was then separated using an external magnet, subjected to multiple washes with ethanol and H_2_O, and dried at 25 ℃ (Scheme 1)^27,28^. To synthesize the Fe_3_O_4_@SiO_2_@A complex, 1 g of the prepared Fe_3_O_4_@SiO_2_ was dispersed in 30 mL EtOH by sonication for 30 min. After that, 3 mmol of 3-aminopropyltriethoxysilane (A) was introduced into the reaction container and stirred under reflux conditions for 24 h. Following the completion of the reaction, the Fe_3_O_4_@SiO_2_@A product was isolated using a magnet and subsequently cleansed with ethyl acetate and EtOH, before being dried at 50 °C in an oven for 15 h. The initial step in the preparation of Fe_3_O_4_@SiO_2_@A@T involved dispersing 1 g of Fe_3_O_4_@SiO_2_@A samples in 20 mL of toluene. Subsequently, 2.5 mmol of trimethylamine (Et_3_N) and 2.5 mmol of 1,3,5-trichloro triazine were added as a cross-linking reagent to the reaction mixture, which was then refluxed for 24 h. The Fe_3_O_4_@SiO_2_@A@T were subsequently separated with the help of an external magnet, cleaned with EtOH, and dried. In the continuation of the nanocatalyst synthesis, Fe_3_O_4_@SiO_2_@A@T (1 g) was reacted with tris(hydroxymethyl)amino methane (0.6 g) and Et_3_N (1.5 mmol) in dry toluene. The mixture was stirred for 24 h at a temperature of 70 °C. Following this duration, the separation, washing, and drying process was carried out as the final step. Finally, to prepare Fe_3_O_4_@SiO_2_@A@TT-Pd organometallic catalytic, a mixture of Fe_3_O_4_@SiO_2_@A@TT (1.0 g), Palladium (II) acetate (2.5 mmol) and 50 ml ethanol was added into the flask, and it was stirred at reflux condition for 24 h. Also, 2 mmol of NaBH_4_ was added to the reaction mixture and stirred for 4 h. After the completion of the reaction, the Fe_3_O_4_@SiO_2_@A-TT-Pd catalyst was separated and washed with H_2_O and ethanol and dried in vacuum.

### Aromatic ethers formation catalyzed by Fe_3_O_4_@SiO_2_@A-TT-Pd

**Scheme 2:**
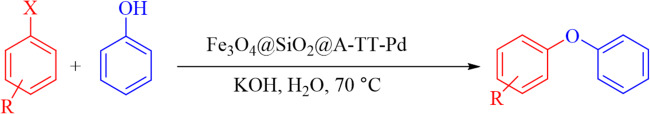
Synthesis of diaryl ether derivatives using Fe_3_O_4_@SiO_2_@A-TT-Pd.

Aryl halide (1 mmol), KOH (5 mmol), phenol (1 mmol), and Fe_3_O_4_@SiO_2_@A-TT-Pd (30 mg) were stirred in H_2_O at 70 °C and the progression of the reaction was seen by TLC. After completing the reaction, the reaction mixture was cooled to room temperature. Subsequently, water was added to dilute the mixture, and the residual catalyst was removed using an external magnet and then rinsed with ethyl acetate. The resulting solution was filtered and then separated into ethyl acetate and water layers. The Na_2_SO_4_ (2 g) was used to dry the solution, after which the solvent was evaporated, yielding pure ether derivatives (Scheme 2).

### A general procedure for the oxidation of sulfides

**Scheme 3:**
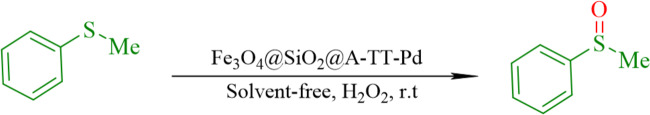
Oxidation of sulfides catalyzed by Fe_3_O_4_@SiO_2_@A-TT-Pd.

A combination of sulfide (2 mmol) and H_2_O_2_ 33% (0.3 mL) was poured into the round-bottomed flask containing Fe_3_O_4_@SiO_2_@A-TT-Pd (0.02 g). The mixture was stirred at room temperature, and the progress of the reaction was monitored by TLC. At the end of the reaction, the catalyst was removed by a magnet, and the products were extracted by water and ethyl acetate. The organic solvents were dried over anhydrous Na_2_SO_4_. Then, the organic solvent was evaporated, and pure products were obtained in high yields (Scheme 3).

### Selected NMR data

Oxydibenzene:^1^H NMR (400 MHz, DMSO): δ_H_ = 7.04 (m, 5 H), 6.83 (m, 5 H) ppm.

1-Methoxy-4-phenoxybenzene:^1^H NMR (400 MHz, DMSO): δ_H_ = 7.47 (d, 1 H), 7.34 (d, 5 H), 7.18 (s, 3 H), 7.18 (s, 3 H) ppm.

1-Nitro-4-phenoxybenzene:^1^H NMR (MHz, DMSO): δ_H_ = 7.95 (d, 3 H), 7.39 (d, 4 H), 7.07 (s, 2 H) ppm

(Sulfinylbis(methylene))dibenzene:^1^H NMR (400 MHz, DMSO): δ_H_ = 7.56 (d, 5 H), 7.28 (d, 5 H), 4.07 (s, 4 H) ppm.

(Ethylsulfinyl)ethane:^1^H NMR (400 MHz, DMSO): δ_H_ = 2.71 (m, 4 H), 1.14 (d, 6 H) ppm.

(Benzylsulfinyl)benzene:^1^H NMR (400 MHz, DMSO): δ_H_ = 2.34 (m, 10 H), 4.17 (d, 2 H) ppm.

#### Catalyst characterizations

**Fig. 1:**
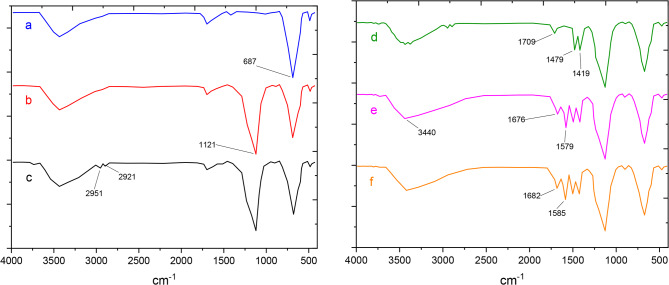
Comparative study of FT-IR spectra of (**a**) Fe_3_O_4_, (**b**) Fe_3_O_4_@SiO_2_, (**c**) Fe_3_O_4_@SiO_2_@A, Fe_3_O_4_@SiO_2_@A@T, (**d**) Fe_3_O_4_@SiO_2_@A@TT, (**e**) Fe_3_O_4_@SiO_2_@A-TT-Pd.

The FTIR spectra of Fe_3_O_4_, Fe_3_O_4_@SiO_2_, Fe_3_O_4_@SiO_2_@A, Fe_3_O_4_@SiO_2_@A@T, Fe_3_O_4_@SiO_2_@A@TT, and Fe_3_O_4_@SiO_2_@A-TT-Pd are shown in Fig. 1. A sharp peak at 678 cm^−1^ in the FT-IR spectrum of bare Fe_3_O_4_ is related to the Fe–O vibrations (Fig. 1a). After encapsulation of Fe_3_O_4_ MNPs, one new peak appears at 1121 cm^−1^ in the FTIR spectrum, which is related to the stretching vibration of Si–O-Si, which is not indicated in the FTIR spectrum of bare Fe_3_O_4_. Furthermore, the stretching vibration of the surface O–H appeared as a broad peak above 3400 cm^−1^ in the FT-IR spectra (Fig. 1b). The surface modification of Fe_3_O_4_@SiO_2_ by APTMS was confirmed by the emergence of multiple new peaks at > 3000 cm^−1^, corresponding to the vibration of aliphatic CH_2_ groups in the propyl chain. This characteristic was not observed in the FT-IR spectrum of Fe_3_O_4_@SiO_2_ (Fig. 1c). After modifying the nanoparticles with cyanuric chloride, three bands at 1709, 1479, and 1419 cm^−1^ appeared, possibly corresponding to the aromatic ring of cyanuric chloride (Fig. 1d). Following the immobilization of tris(hydroxymethyl)amino methane on nanoparticles, a distinctive peak emerges at 1579 cm^−1^, potentially indicative of the bending vibration of N–H groups. Also, the peak appearing in the region of 2500–3400 is related to the hydroxy ligand group of tris(hydroxymethyl)amino methane (Fig. 1e). More importantly, the change in the intensity of the peaks of Fe_3_O_4_@SiO_2_@A@TT-Pd confirms the coordination of the nitrogen atom of the amino groups to Pd (Fig. 1f).

**Fig. 2:**
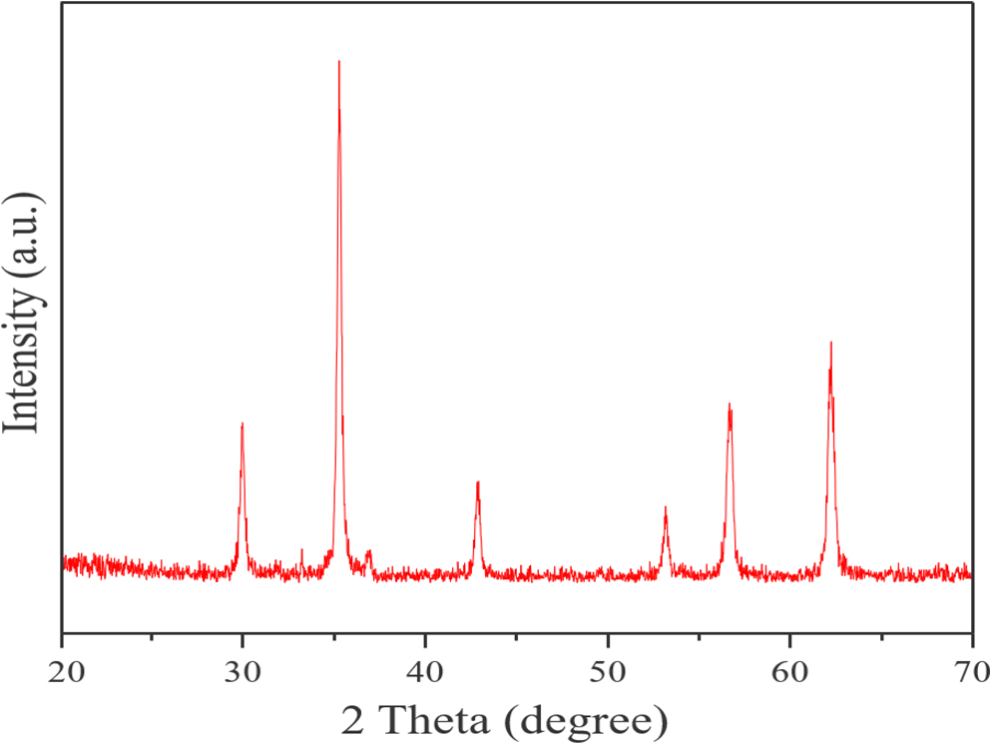
XRD spectrum of Fe_3_O_4_@SiO_2_@A-TT-Pd.

The diffraction patterns of Fe_3_O_4_@SiO_2_@A-TT-Pd are depicted in Fig. 2. A study using powder X-ray diffraction (XRD) was conducted to analyze the phase behavior and crystallinity of the catalyst. The initial phases in the 2θ region up to 30°, 36°, 45°, 54°, 57°, and 64° were identified as the (2 2 0), (3 1 1), (4 0 0), (4 2 2), (5 1 1), and (4 4 0) planes of the highly crystalline Fe_3_O_4_@SiO_2_@A-TT-Pd nanostructure. The XRD pattern of the catalyst indicates that the Fe_3_O_4_ phase remained unchanged following the modifications with a distinct organic functional group (Fig. 2).

**Fig. 3:**
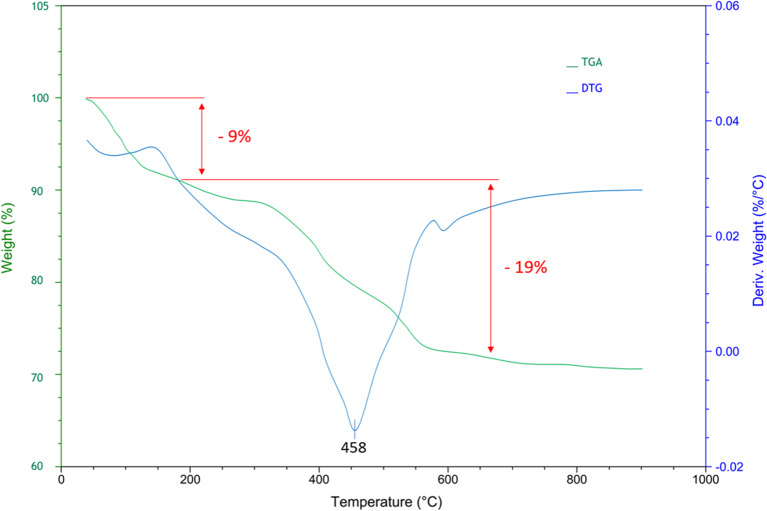
TGA-DTG curve of Fe_3_O_4_@SiO_2_@A-TT-Pd.

The TGA-DTG curve of Fe_3_O_4_@SiO_2_@A-TT-Pd indicated a weight loss of 9% below 200 °C which corresponds to desorption of physically adsorbed solvents. The most significant weight loss, about 19%, occurs within the temperature range of 200 to 600 °C, and it is associated with the elimination of organic compounds from Fe_3_O_4_ (see Fig. 3). which shows the thermal stability of the mentioned catalyst up to a temperature of 600 °C. Also, the successful attachment of A-TT-Pd to the surface of Fe_3_O_4_ MNPs was confirmed through the results of TGA analysis.

**Fig. 4:**
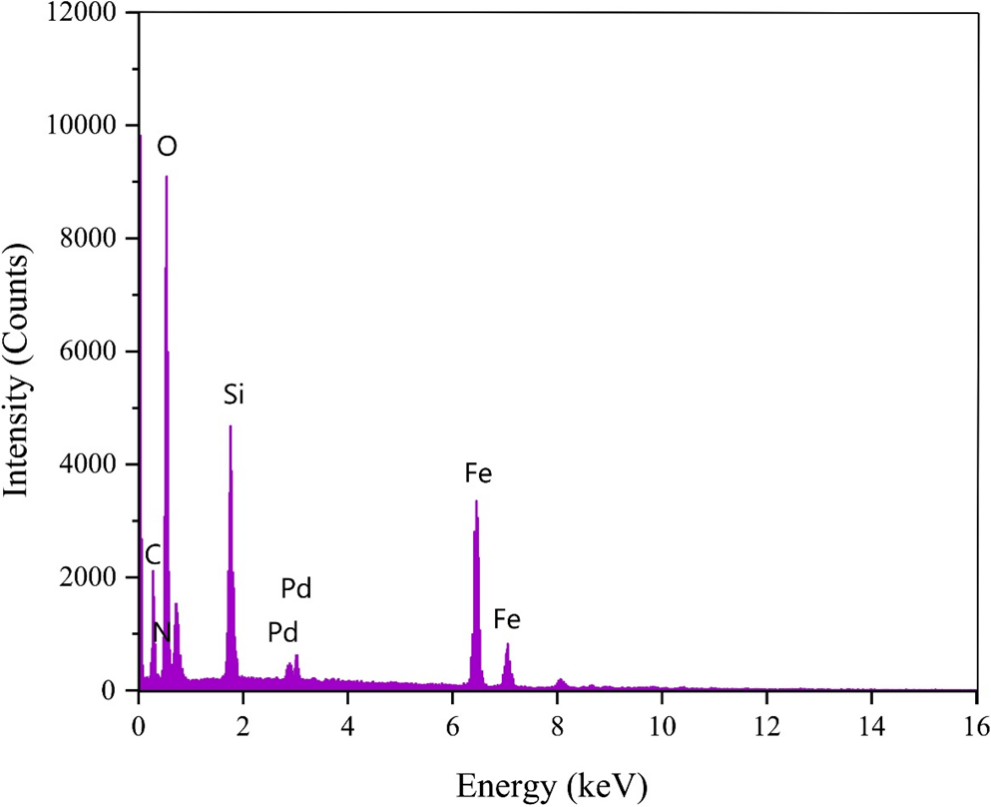
EDS analysis of Fe_3_O_4_@SiO_2_@A-TT-Pd.

EDX is one of the best approaches to determining elements present in nanoparticles and the purity of nanoparticles (Fig. 4). Figure 4 illustrates the EDX spectrum of Fe_3_O_4_@SiO_2_@A-TT-Pd MNPs, confirming the presence of C, N, Fe, O, Si, and Pd in the catalyst and providing evidence for the successful synthesis of nanoparticles.

**Fig. 5:**
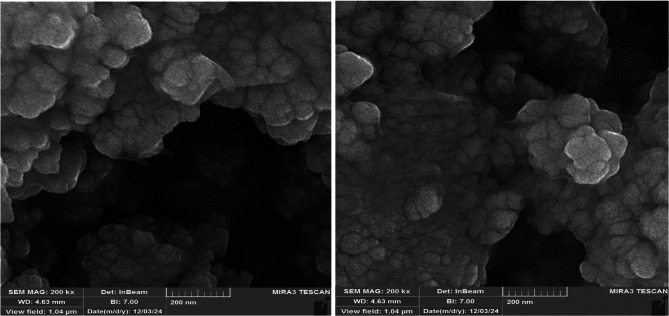
SEM images of Fe_3_O_4_@SiO_2_@A-TT-Pd.

The SEM analysis revealed the morphology and dimensions of Fe_3_O_4_@SiO_2_@A-TT-Pd. The resulting SEM image illustrated that the nanoparticles were spherical and within the nano-size range (Fig. 5).

**Fig. 6:**
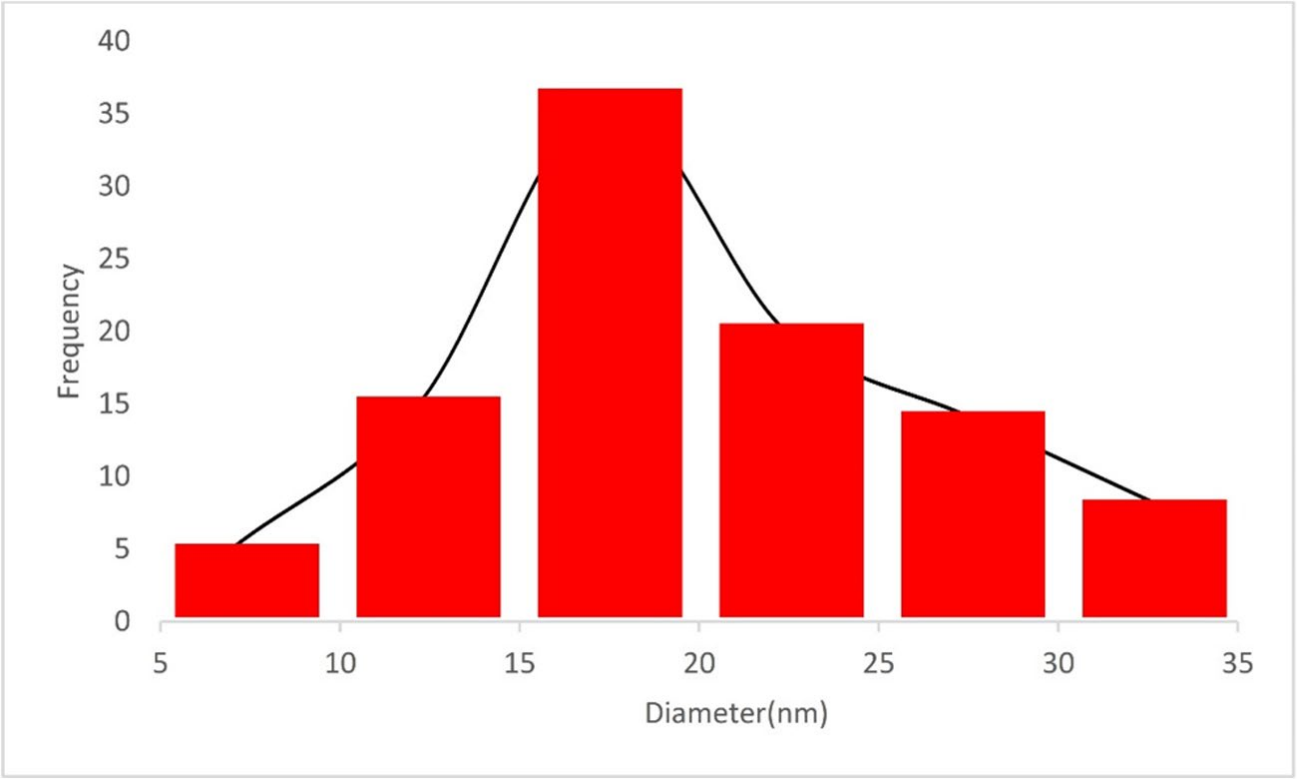
Particle size distribution histogram of Fe_3_O_4_@SiO_2_@A-TT-Pd.

To demonstrate the particle size distribution of these catalysts, Fig. 6 displays the histogram of particle sizes extracted from SEM images. As shown, the particle size of Fe_3_O_4_@SiO_2_@A-TT-Pd shows homogeneous diameters in the obtained histogram SEM images.

**Fig. 7:**
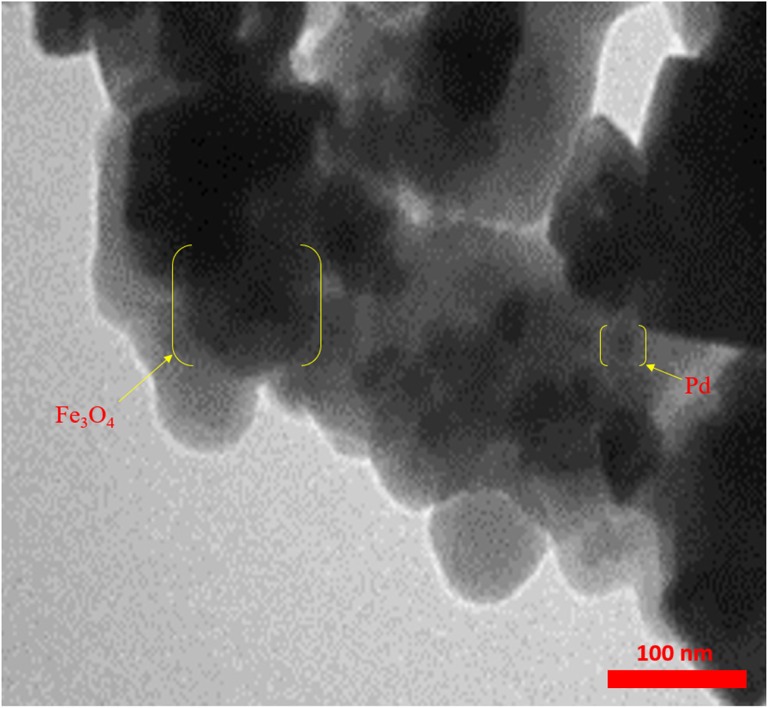
TEM images of Fe_3_O_4_@SiO_2_@A-TT-Pd.

Also, the morphology of the Fe_3_O_4_@SiO_2_@A-TT-Pd was investigated by TEM, as shown in Fig. 7. The images revealed the modified Fe_3_O_4_ nanoparticles with TT-Pd as an organic shell covered. Images from scanning electron microscopy reveal that the catalyst particles are within the nanometer range and exhibit a spherical structure. This was further confirmed by the data obtained from transmission electron microscopy images (Fig. 7).

Furthermore, ICP-OES analysis was conducted to determine the quantity of Pd in Fe_3_O_4_@SiO_2_@A-TT-Pd. According to the analysis, the catalyst was determined to contain 2.4 × 10^−4^ mol of Pd per gram based on ICP-OES. The Pd leaching amount after recycling the catalyst was investigated through ICP-OES analysis. Based on this analysis, the reused catalysts contain 2.3 × 10^−4^ mol. g^−1^ of Pd, indicating minimal leaching of Pd from the Fe_3_O_4_@SiO_2_@A-TT-Pd framework.

**Fig. 8:**
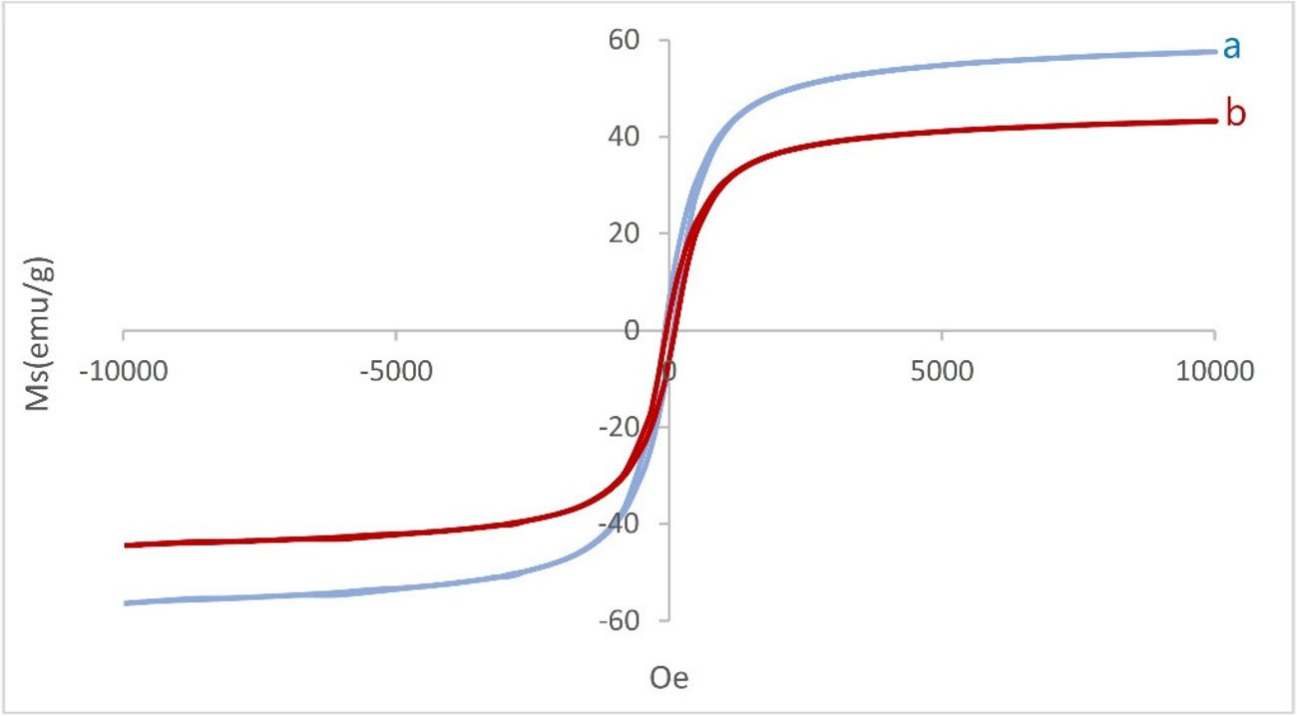
VSM curves of (**a**) Fe_3_O_4_ (**b**) Fe_3_O_4_@SiO_2_@A-TT-Pd.

The magnetic behavior of Fe_3_O_4_ (Fig. 8a), and Fe_3_O_4_@SiO_2_@A-TT-Pd (Fig. 8b) was investigated using VSM techniques. As expected, the decrease in saturation magnetization from about 61 emu/g to about 43 emu/g, is related to the newly coated layer (Fig. 8).

#### Catalytic studies

The catalytic activity of Fe_3_O_4_@SiO_2_@A-TT-Pd was studied in the C-O coupling reaction for producing diaryl ether derivatives. In the production of diaryl ethers, the pairing of phenol with iodobenzene using the catalytic potential of Fe_3_O_4_@SiO_2_@A-TT-Pd has been selected as a model reaction to determine the optimized conditions. Initially, the pattern reaction was tested without Fe_3_O_4_@SiO_2_@A-TT-Pd, resulting in the pattern reaction not proceeding. Then, the pattern reaction was carried out using the variant value of the catalyst which was completed with 99% of yield when 0.02 g of Fe_3_O_4_@SiO_2_@A-TT-Pd was used (Table 1). The reaction pattern was studied under a wide range of temperatures, with a focus on the effects of different solvents and bases. The best results for diaryl ether synthesis were achieved using H_2_O as the solvent and KOH as the base at 70 ºC, as shown in Table 1.

**Table 1:** Optimization of different parameters for the reaction of phenol with iodobenzene.


Entry	Catalyst (g)	Solvent	Base	Temperature (˚C)	Time (min)	Yield (%)^a^
**1**	-	H_2_O	KOH	70	3 days	N. R
**2**	0.008	H_2_O	KOH	70	30	40
**3**	0.01	H_2_O	KOH	70	30	78
**4**	0.015	H_2_O	KOH	70	30	95
**5**	0.02	H_2_O	KOH	70	30	99
**6**	0.025	H_2_O	KOH	70	30	99
**7**	0.02	PEG-400	KOH	70	30	40
**8**	0.02	EtOAc	KOH	70	30	75
**9**	0.02	Acetonitrile	KOH	70	30	82
**10**	0.02	EtOH	KOH	70	30	89
**11**	0.02	H_2_O	Li_2_CO_3_	70	30	90
**12**	0.02	H_2_O	Na_2_CO_3_	70	30	90
**13**	0.02	H_2_O	Cs_2_CO_3_	70	30	88
**14**	0.02	H_2_O	NaHCO_3_	70	30	75
**15**	0.02	H_2_O	K_2_CO_3_	70	30	45
**16**	0.02	H_2_O	KOH	25	30	Trace
**17**	0.02	H_2_O	KOH	50	30	45
**18**	0.02	H_2_O	KOH	Reflux	30	99

^a^ Isolated yield.

The optimizing conditions mentioned were explored for various aryl halide derivatives to broaden the catalytic scope of Fe_3_O_4_@SiO_2_@A-TT-Pd (Table 2). Aryl halide derivatives with different functional groups, whether electron-withdrawing or electron-donating in nature, were effectively coupled with phenol in high yields using this catalyst. As indicated in Table 2, aryl iodides exhibit a higher reaction rate compared to aryl bromides, while aryl chlorides demonstrate the lowest reaction rate when coupling phenol using the Fe_3_O_4_@SiO_2_@A-TT-Pd catalyst. This suggests that the C–Cl bond is stronger than the C–I bond, as the carbon and chlorine orbitals share similar size, energy, and symmetry, whereas the iodine and carbon orbitals differ in size and energy. Furthermore, the C–I bond is longer and weaker than the C–Cl bond, requiring less energy to break and resulting in a faster coupling rate compared to the shorter C–Cl bond. For instance, the coupling of phenol with 4-nitrobromobenzene surpasses that of 4-nitrochlorobenzene. This pattern is also evident in the coupling of phenol with iodobenzene, bromobenzene, and chlorobenzene using the Fe_3_O_4_@SiO_2_@A-TT-Pd catalyst.

**Table 2:**
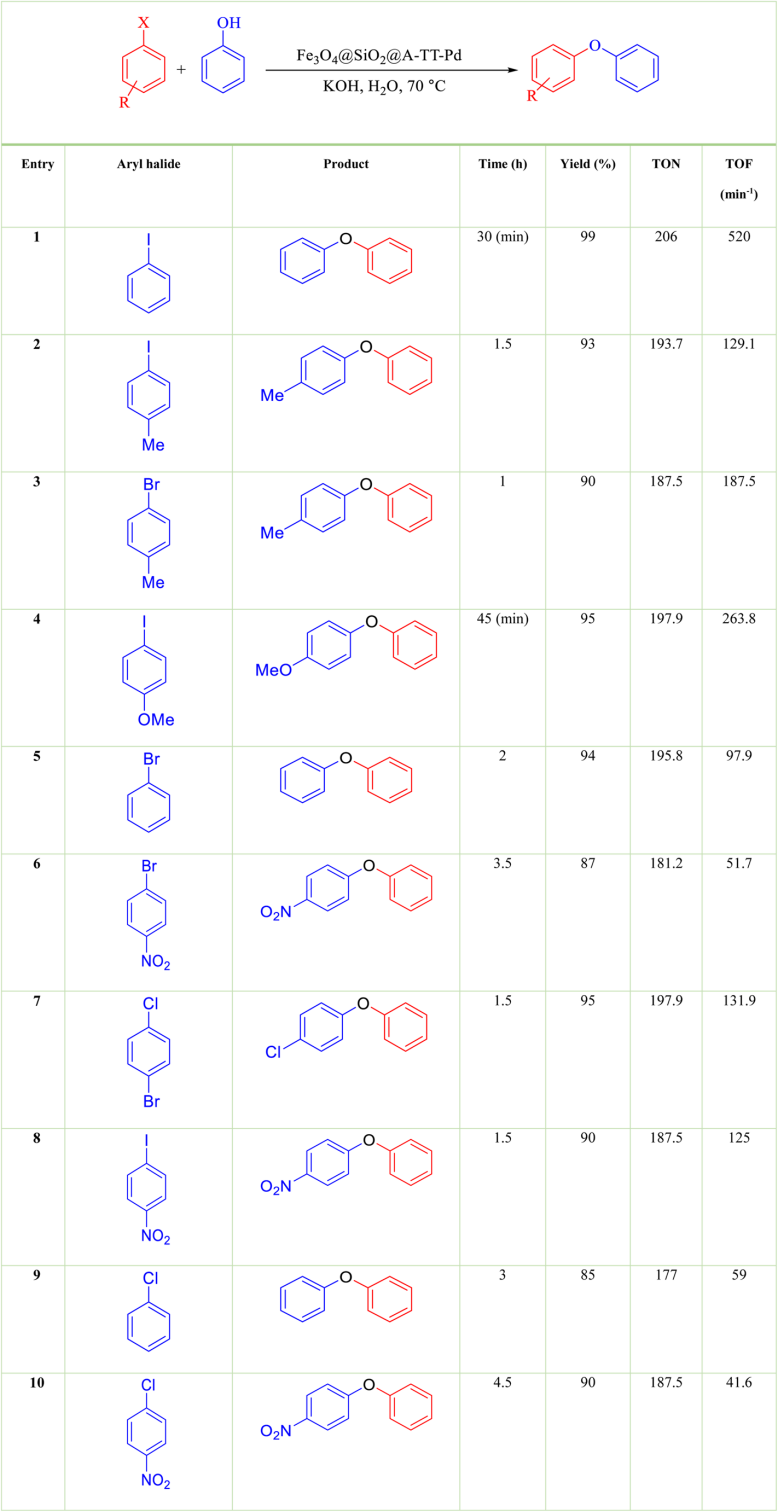
Synthesis of diaryl ether derivatives using Fe_3_O_4_@SiO_2_@A-TT-Pd.

**Scheme 4:**
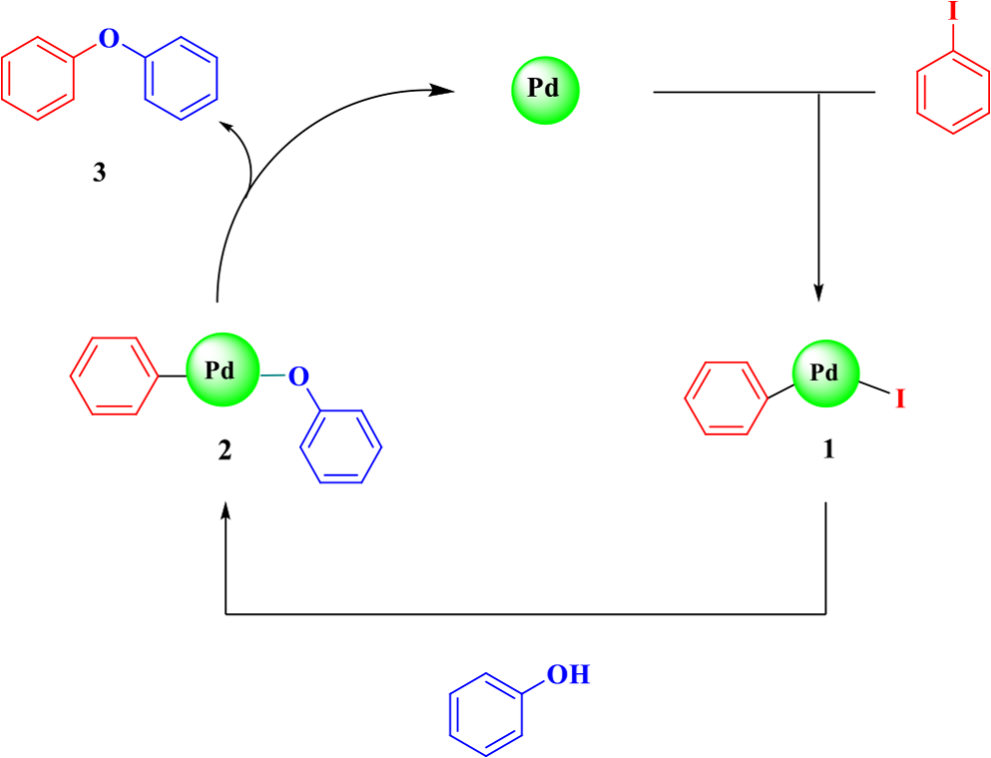
Proposed mechanism for C–O cross-coupling.

The Carbon-Oxygen cross-coupling reaction’s proposed mechanism, as depicted in Scheme 4, is based on previous findings. In the initial step, iodobenzene undergoes oxidative addition with Pd, yielding intermediate (1). Subsequently, intermediate (1) engages with phenol to generate intermediate (2), which ultimately undergoes reductive elimination to yield ether while liberating the Pd nanoparticle.

To optimize the reaction conditions, we investigated the oxidation process of methyl phenyl sulfide as a representative compound using H_2_O_2_ under different reaction parameters, including time and product yield (see Table 3). As shown in Table 3, the reaction was incomplete in the absence of Fe_3_O_4_@SiO_2_@A-TT-Pd even after 12 h. Under solvent-free conditions at room temperature, utilizing a catalytic amount of Fe_3_O_4_@SiO_2_@A-TT-Pd (0.01 g), H_2_O_2_ was determined to be the optimal reagent for the complete conversion of methyl phenyl sulfide to methyl phenyl sulfoxide.

**Table 3:** Optimizing reaction conditions for oxidation of methyl phenyl sulfide in the presence of Fe_3_O_4_@SiO_2_@A-TT-Pd.


Entry	Catalyst(g)	Solvent	H_2_O_2_ (mg)	Time (min)	Yield
1	-	Solvent-free	0.3	12 h	Trace
2	0.005	Solvent-free	0.3	15	60
3	0.07	Solvent-free	0.3	15	84
4	0.01	Solvent-free	0.3	15	98
5	0.02	Solvent-free	0.3	15	98
6	0.01	H_2_O	0.3	15	50
7	0.01	PEG	0.3	15	49
8	0.01	EtOH	0.3	15	47
9	0.01	DMF	0.3	15	80
10	0.01	Solvent-free	0.2	15	90
11	0.01	Solvent-free	0.4	15	96

The generality of this approach has been demonstrated by facile oxidation of aryl, cyclic, benzylic, and linear as shown in Table 4. The sulfoxides were quickly obtained with high yields. To demonstrate the chemoselectivity of the protocol, sulfides containing oxidation-prone and acid-sensitive functional groups such as CHO, OH, and CO_2_CH_3_ were used in the sulfoxidation reaction. Importantly, these functional groups remained unaffected during the sulfide to sulfoxide conversion, as shown in Table 4. Catalyst evaluation heavily relies on selectivity, which plays a crucial role in determining their effectiveness. Chemoselectivity specifically refers to the reactivity of a functional group when other susceptible functional groups are present and subject to the same reaction. An example of this is the oxidation of sulfide in the presence of a hydroxyl group, which can also be oxidized to form a carbonyl. The chemoselectivity of Fe_3_O_4_@SiO_2_@A-TT-Pd was investigated in the oxidation of 2-phenylthioethanol. This catalyst shows good chemoselectivity in synthesizing sulfoxides in the oxidation of 2-phenylthioethanol (Table 4, entry 9).

**Table 4:**
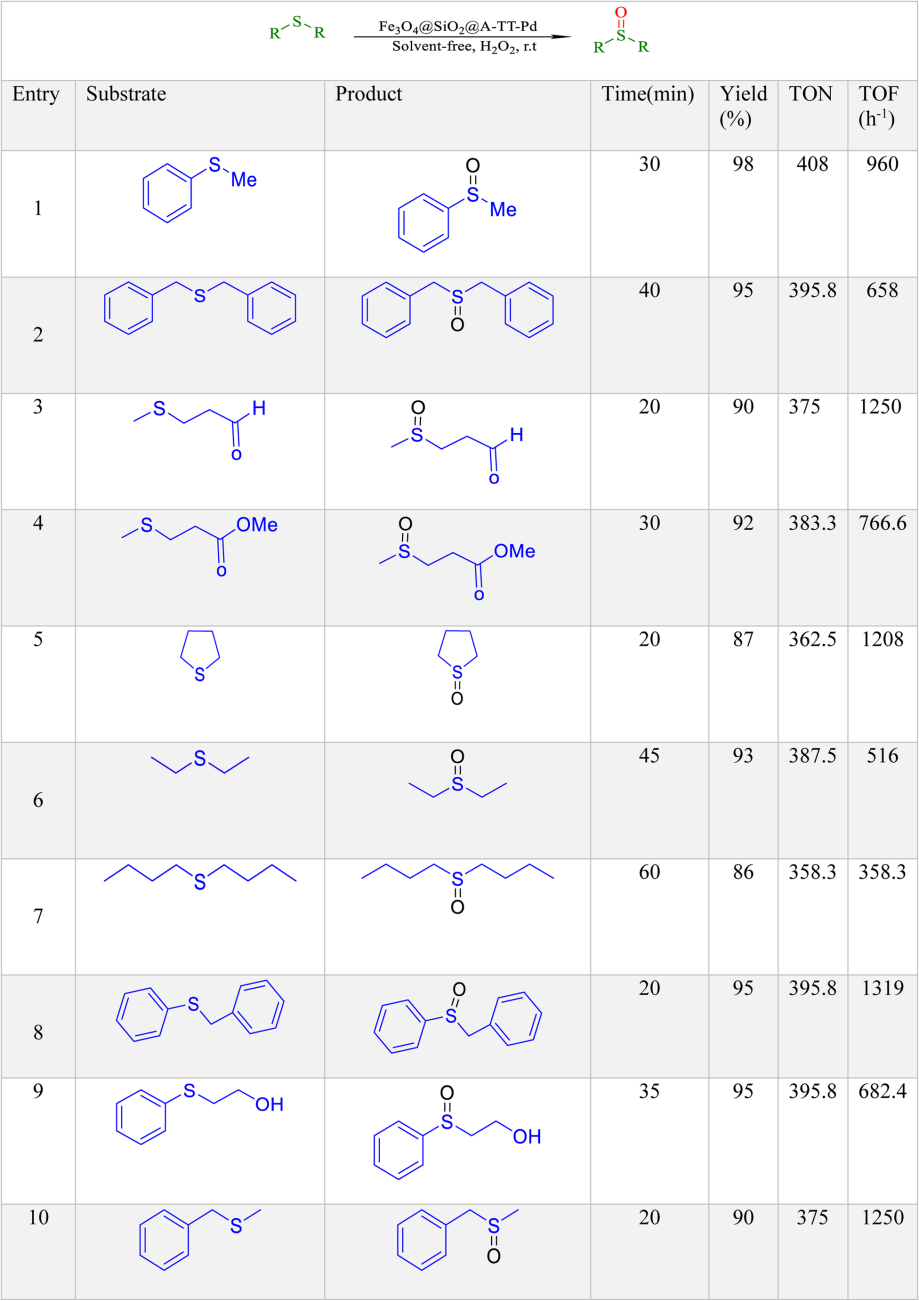
Oxidation of sulfides into sulfoxides in the presence of Fe_3_O_4_@SiO_2_@A-TT-Pd.

**Scheme 5:**
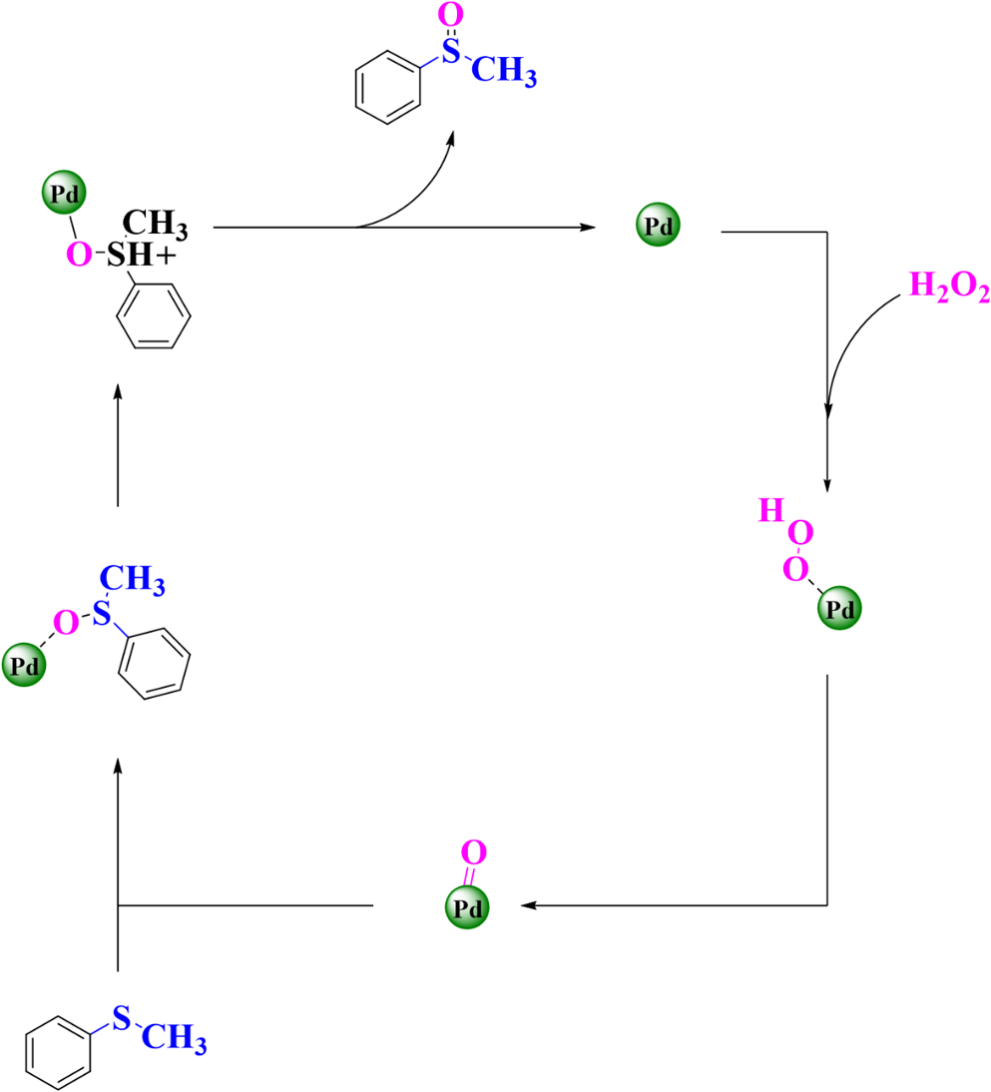
The suggested mechanism for the oxidation of sulfide.

Based on previous studies, a suggested and possible mechanism for the oxidation of sulfides catalyzed by Fe_3_O_4_@SiO_2_@A-TT-Pd has been presented in Scheme 5. In Fe_3_O_4_@SiO_2_@A-TT-Pd, Palladium plays a crucial role as a magnetic nanocatalyst by forming the active oxidant complex. This mechanism facilitates the transfer of oxygen to sulfur, resulting in the formation of sulfoxide.

#### Hot filtration

In order to determine any leaching of palladium in the reaction mixture and to demonstrate the heterogeneous nature of Fe_3_O_4_@SiO_2_@A-TT-Pd catalyst, a hot filtration test was conducted during the synthesis of diaryl ethers of iodobenzene with phenol. According to the study, the yield of the product reached 58% in half of the reaction time. Subsequently, the experiment was replicated, and at the midpoint of the reaction, the catalyst was separated, allowing the filtrate to continue reacting. The yield at this stage amounted to 60%, thus confirming the absence of palladium leaching.

**Fig. 9:**
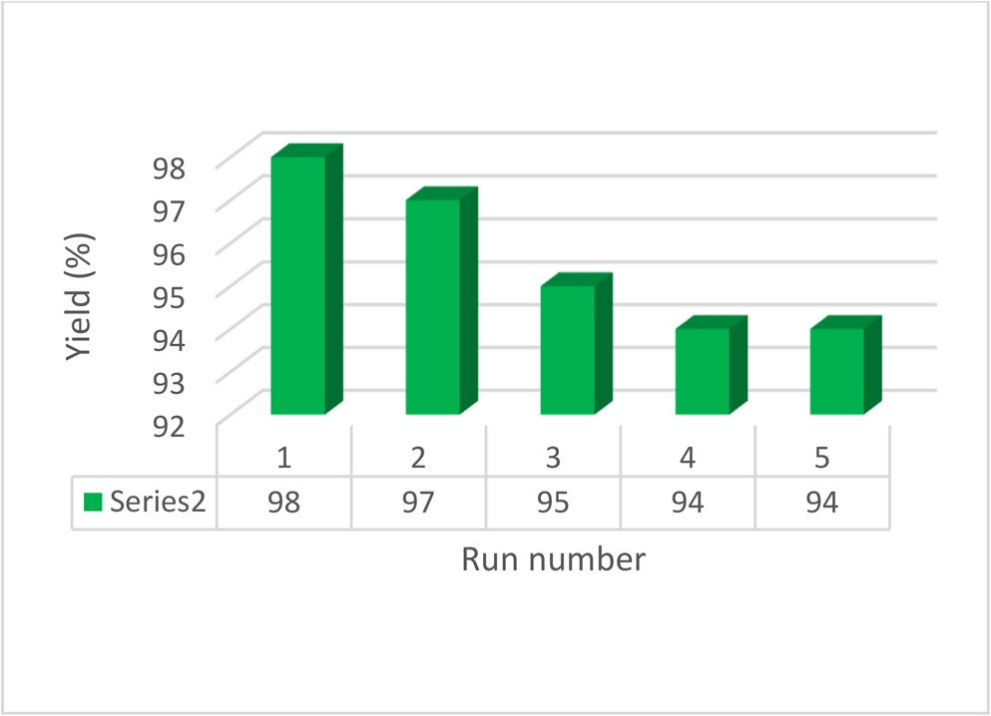
Recyclability of Fe_3_O_4_@SiO_2_@A-TT-Pd in the oxidation of sulfide.

The ability to reuse catalysts is a crucial advantage, making them valuable for commercial use. We discovered that Fe_3_O_4_@SiO_2_@A-TT-Pd was rapidly recovered and displayed outstanding recyclability. To explore this, we examined the catalyst’s recyclability in the oxidation of methyl phenyl sulfide. Following the reaction, the catalyst was isolated, rinsed with ethanol to eliminate any remaining product, and dried. Fresh substrates were then introduced to the remaining catalyst for the subsequent reaction. As depicted in Fig. 9, this catalyst can be reused for up to 5 cycles without experiencing significant loss of catalytic activity or Pd leaching.

#### Comparison of catalyst

To explain the catalytic activity of Fe_3_O_4_@SiO_2_@A-TT-Pd, we analyzed the outcomes of methylphenyl sulfide oxidation using this catalyst and compared them with previously documented methods (Table 5). Notably, in contrast to other catalysts, the preparation of Fe_3_O_4_@SiO_2_@A-TT-Pd is straightforward using cost-effective and readily available materials, and it can be reused up to five times with no significant decline in activity. This catalyst results in a favorable reaction time and higher yield when compared to others. As a result, this novel catalyst demonstrates comparable or even superior characteristics in terms of cost, non-toxicity, stability, and ease of separation.

**Table 5:** Comparing Fe_3_O_4_@SiO_2_@A-TT-Pd for oxidation of methyl phenyl sulfide with previously reported procedures.

Entry	Substrate	Catalyst	Time (min)	Yield (%)	Ref.
1	Ph–SCH_3_	Ni-dithizone@boehmite	80	96	^29^
2	Ph–SCH_3_	Cu-SB-APT@MCM-41	45	91	^30^
3	Ph–SCH_3_	Polymer-anchored Cu (II)	180	90	^31^
4	Ph–SCH_3_	VO_2_F(dmpz)_2_	300	95	^32^
5	Ph–SCH_3_	SiO_2_–W_2_–Im	150	91	^33^
6	Ph–SCH_3_	Fe_3_O_4_@SiO_2_@A-TT-Pd	15	98	This work

## Conclusion

In summary, we have reported Fe_3_O_4_@SiO_2_@A-TT-Pd as a green, efficient, and reusable catalyst for the oxidation of sulfides to sulfoxides and the synthesis of a wide range of diaryl ether derivatives. Both activity and selectivity in Fe_3_O_4_@SiO_2_@A-TT-Pd are much higher than other previous catalysts. These protocols offer numerous benefits, including the utilization of readily available, cost-effective, environmentally friendly, and chemically stable materials, as well as straightforward operation and high to excellent yields. The simple procedure for preparation, use of non-toxic solvents, short reaction time, excellent tolerance of our method towards different functional groups, and the ability to recycle and reuse the catalyst using an external magnet up to five times with only a minor decrease in product yields are several advantages of this method. Furthermore, the Fe_3_O_4_@SiO_2_@A-TT-Pd is more cost-effective and environmentally friendly due to its low Pd leaching.

## Supplementary Information

Supplementary Material 1.

## Data Availability

All data generated or analyzed during this study are included in this published article and its supplementary information files.
