# Supplementary material for: In situ decorated pd NPs on Triazin-encapsulated Fe3O4/SiO2-NH2 as magnetic catalyst for the synthesis of diaryl ethers and oxidation of sulfides
Source: Sci Rep. 2024 Oct 25;14:25261. doi: 10.1038/s41598-024-75681-x (PMC11502755; doi:10.1038/s41598-024-75681-x)
Supplement: Supplementary file 1 — Supplementary Material 1. [file 41598_2024_75681_MOESM1_ESM.docx]

**In situ decorated Pd NPs on** **Triazin-encapsulated Fe_3_O_4_/SiO_2_-NH_2_ as magnetic** **catalyst** **for the synthesis of** **diaryl ethers and** **oxidation of sulfides**

Durgesh Singh^1^*, Kamini Singh^2^, Pawan Sharma^3,4^, Yashwantsinh Jadeja^5^, Johar MGM^6^, Priyanka Singh^7^, Kiranjeet Kaur^8^, M. Atif^9^, Mohammed A. El-Meligy^10^, Beneen Husseen^11,12^

^1^Department of Chemistry, School of Chemical Sciences and Technology, Dr.Harisingh Gour Vishwavidyalaya (A Central University), Sagar - 470003, India

^2^Department of Chemistry, Deen Dayal Upadhyay, Gorakhpur University, Gorakhpur 273009, India

^3^Department of Chemistry, School of Sciences, Jain (Deemed-to-be) University, Bengaluru, Karnataka-560069, India

^4^Department of Sciences, Vivekananda Global University, Jaipur, Rajasthan-303012, India

^5^Marwadi University Research Center,Department of Chemistry, Faculty of Science

Marwadi University, Rajkot-360003, Gujarat, India

^6^Management and Science University, Shah Alam, Selangor, Malaysia

^7^NIMS School of Allied Sciences and Technology, NIMS University, Rajasthan, Jaipur, 303121, India

^8^Chandigarh Pharmacy College, Chandigarh Group of colleges-Jhanjeri, Mohali 140307,Punjab, India

^9^Department of Physics and Astronomy, College of Science, King Saud University, P O Box 2455, Riyadh 11451, Saudi Arabia

^10^Applied Science Research Center, Applied Science Private University, Amman, Jordan MEU Research Unit, Middle East University, Amman 11831, Jordan

^11^Medical laboratory technique college, the Islamic University, Najaf, Iraq.

^12^Medical laboratory technique college, the Islamic University of Al Diwaniyah, Al Diwaniyah, Iraq


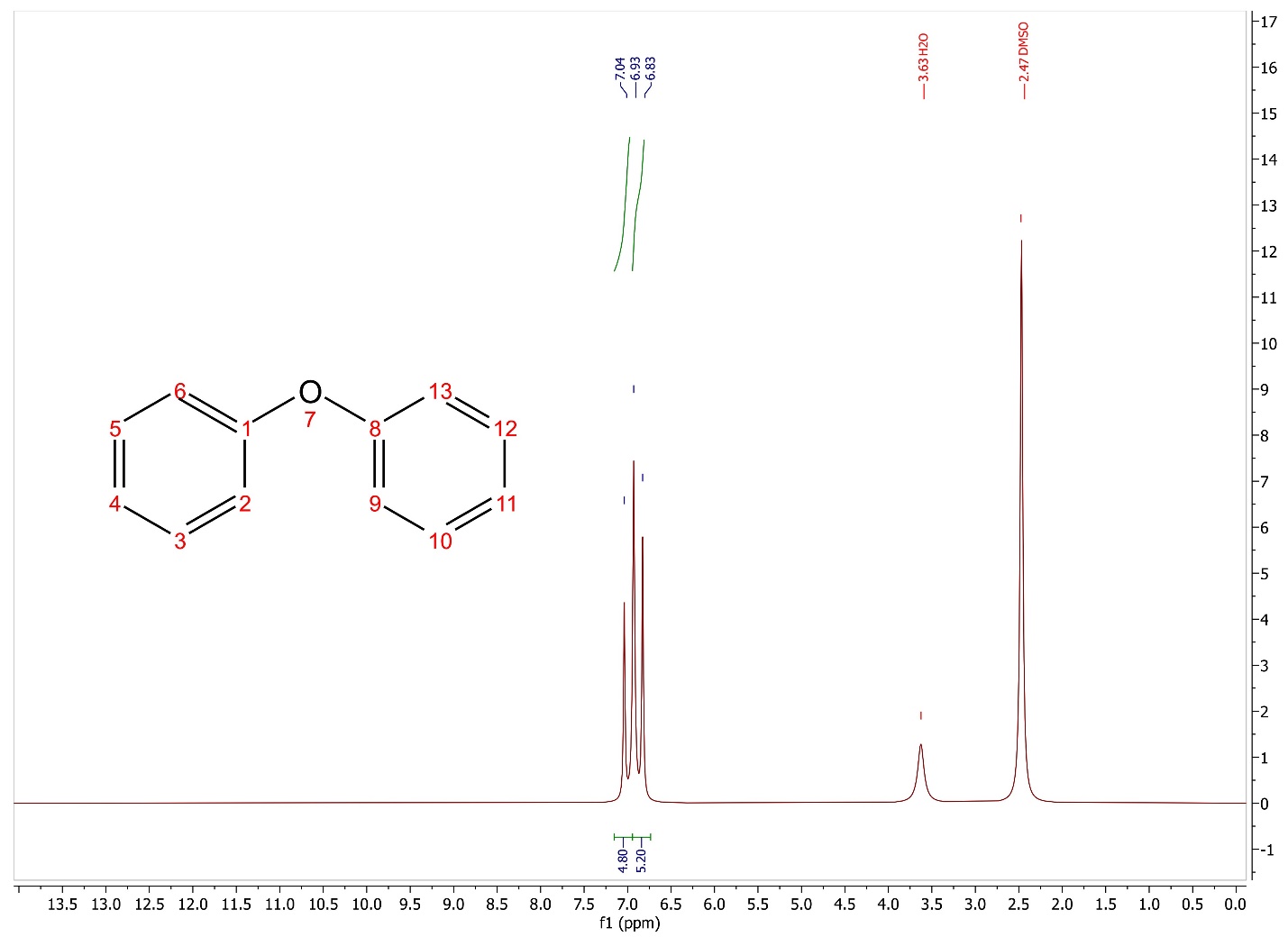


Fig. 1 Oxydibenzene


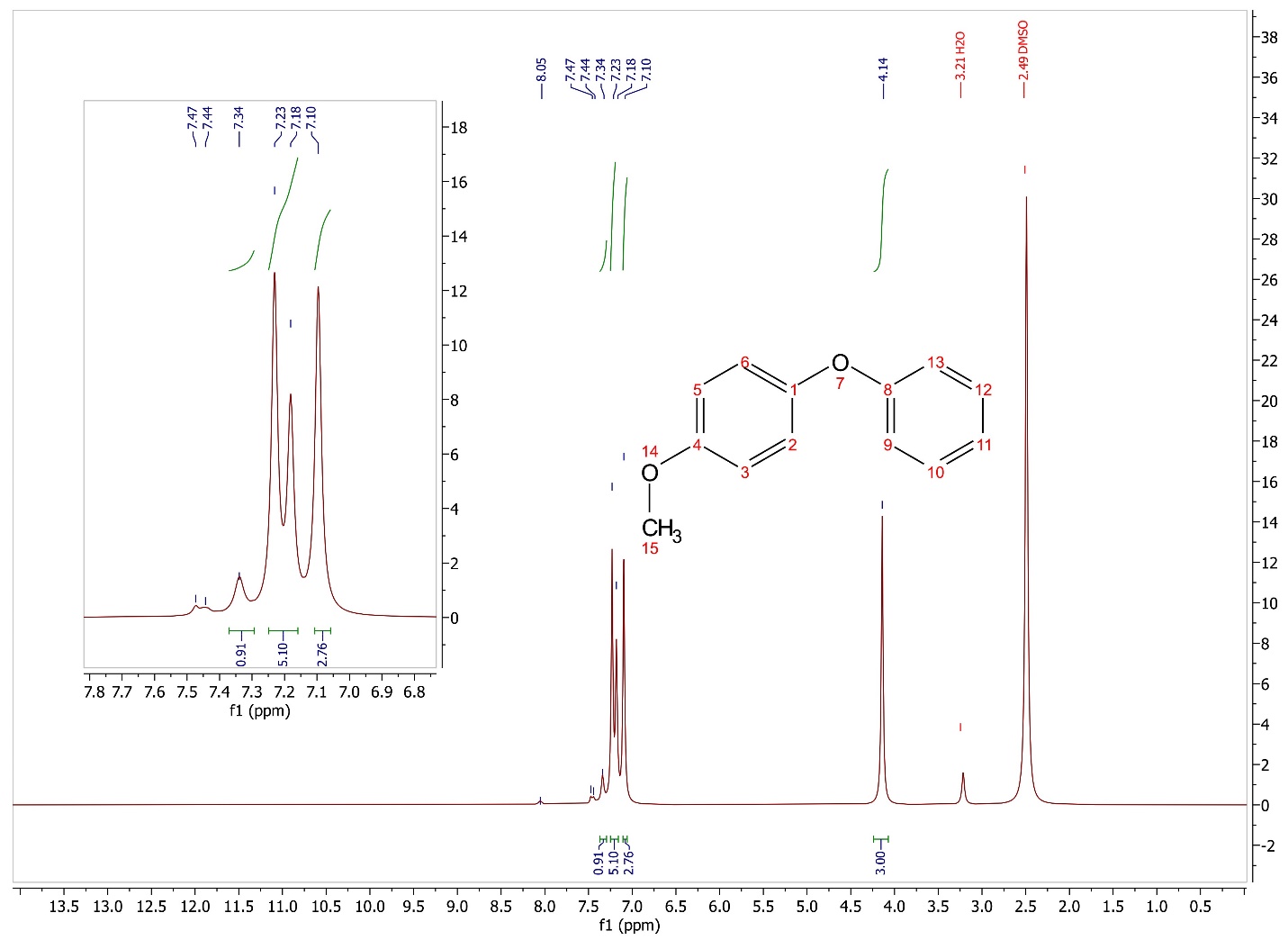


Fig. 2 1-methoxy-4-phenoxybenzene


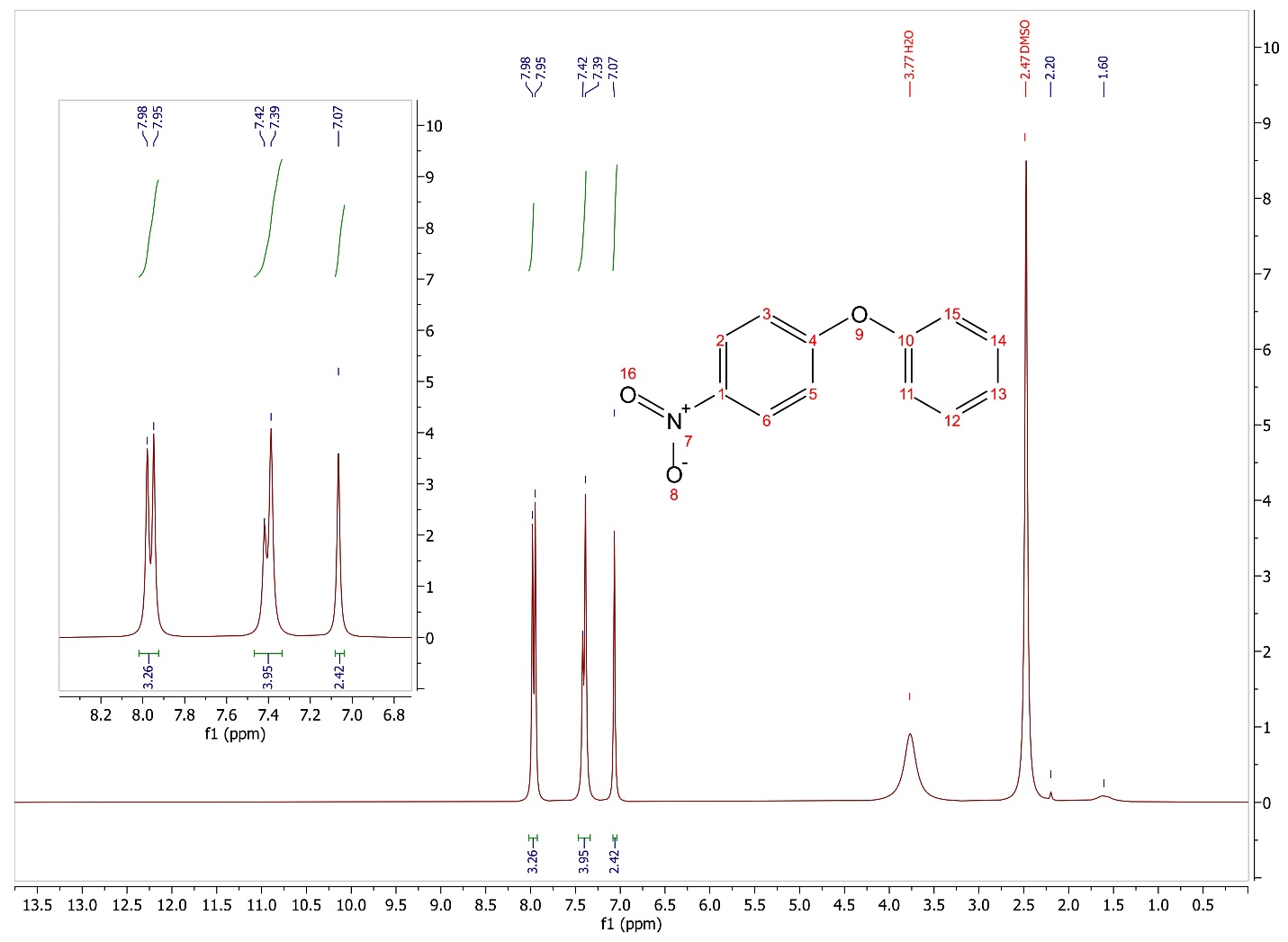


Fig. 3 1-nitro-4-phenoxybenzene


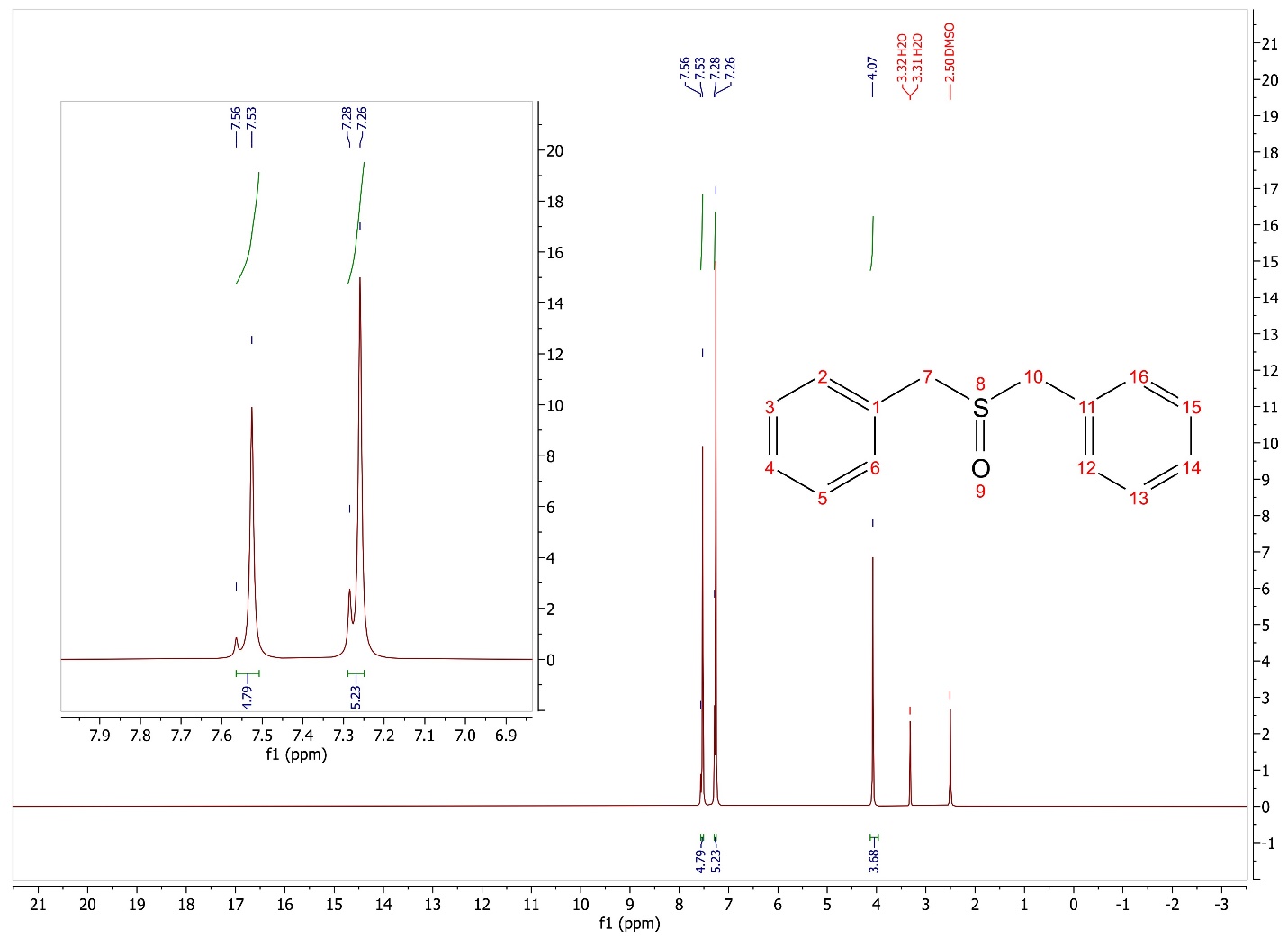


Fig. 4 (sulfinylbis(methylene))dibenzene


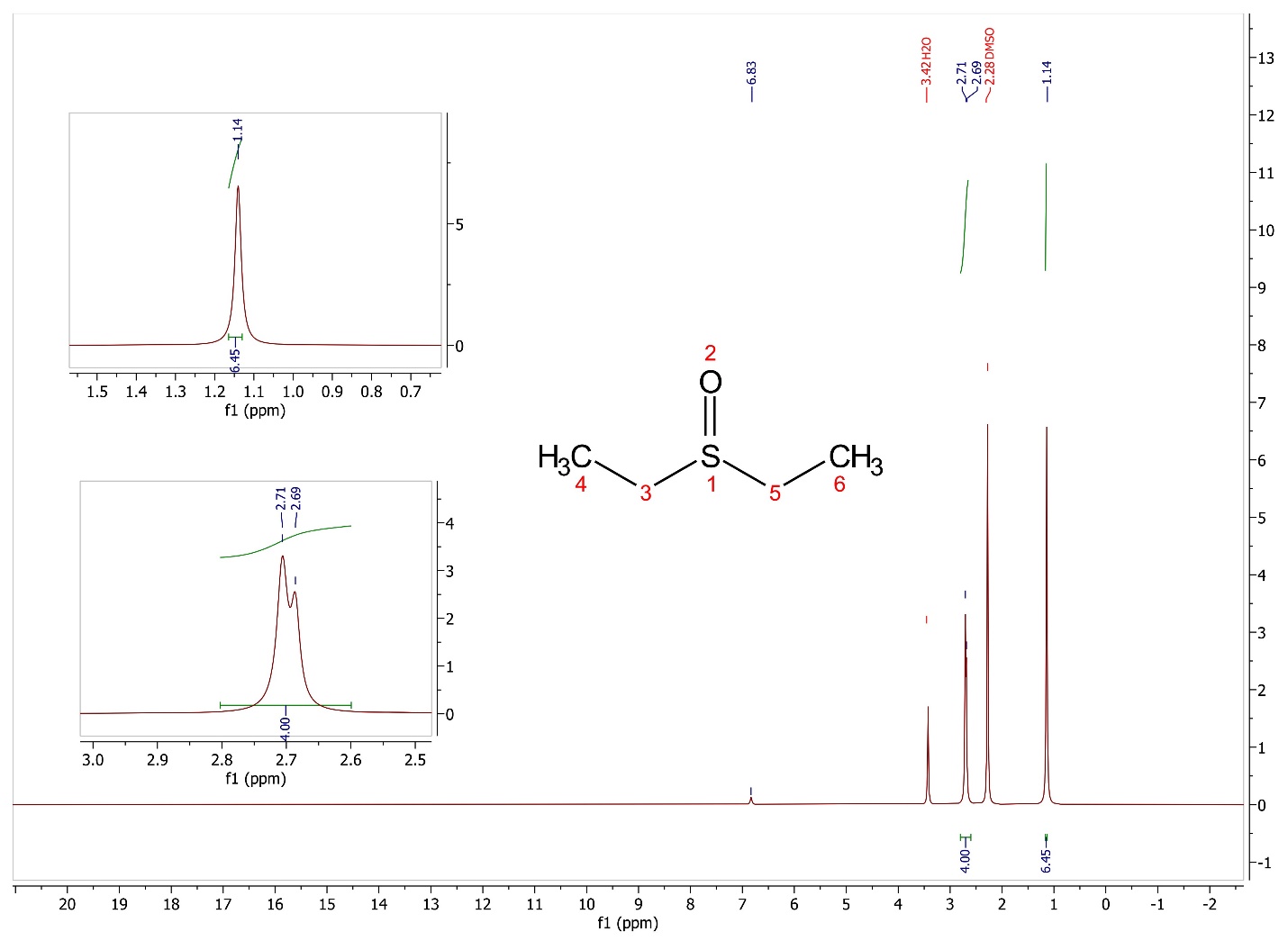


Fig. 5 (ethylsulfinyl)ethane


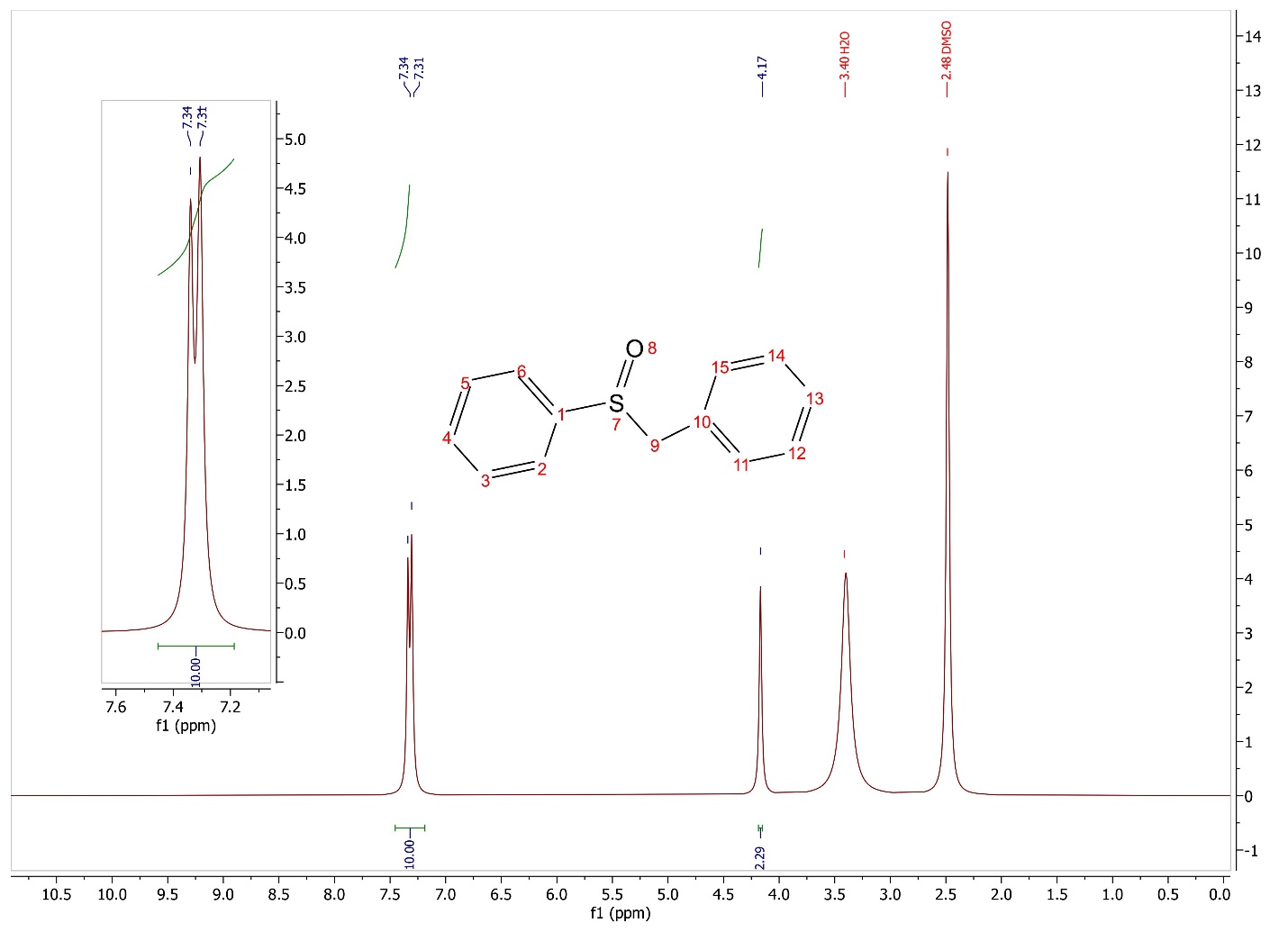


Fig. 6 (benzylsulfinyl)benzene
